# COVID-19 and Pregnancy Outcomes: A Descriptive Study From a Tertiary Hospital in Ras Al Khaimah, UAE

**DOI:** 10.1155/ogi/5252919

**Published:** 2024-12-03

**Authors:** Manal M. Sami, Shatha Al Zuheiri, Nour K. Sabaneh, Mustafa Amir Abdul Latif, Shooq Yousef Al-Blooshi, Mira Osman

**Affiliations:** ^1^Department of Pathology, RAK College of Medical Sciences, Ras Al Khaimah Medical and Health Sciences University, Ras Al Khaimah, UAE; ^2^Obstetrics and Gynecology Department, Abdullah Bin Omran Hospital for Obstetrics and Gynecology, Emirates Health Services, Ras Al Khaimah, UAE; ^3^Clinical Sciences Department, RAK College of Medical Sciences, Ras Al Khaimah Medical and Health Sciences University, Ras Al Khaimah, UAE

**Keywords:** COVID-19, COVID-19 in pregnancy, fetal outcomes, infectious diseases, maternal outcomes, women's health

## Abstract

**Background:** Over 768 million cases of COVID-19 infection have been reported worldwide, with pregnant women being the most vulnerable members of society during such an infectious disease outbreak. In the United Arab Emirates, there are limited studies explaining the effects of COVID-19 on pregnant women and their fetuses. In this study, the maternal and fetal outcomes in pregnant women with COVID-19 in a tertiary maternal hospital, United Arab Emirates, were examined.

**Materials and Methods:** A descriptive study was conducted in a tertiary hospital for Obstetrics and Gynecology in Ras Al Khaimah, UAE. The study included all pregnant women who tested positive for COVID-19 infection from April 2020 to September 2021.

**Results:** The study revealed that a higher number of COVID-19-infected pregnant patients presented in their third trimester (69.1%). The comorbidity of body mass index (BMI) had the most effect on the severity/hospitalization status of the patients (*p*=0.018). In the nonhospitalized group, fever was the most common symptom (26%), whereas in the hospitalized group, cough was the most common symptom (94%). Emergency cesarean delivery was found to be significant (*p*=0.0007) in hospitalized patients. COVID-19 pneumonia was the prevailing adverse maternal outcome. NICU admission and prematurity were the most frequent neonatal outcomes.

**Conclusions:** In conclusion, our findings show that adverse maternal outcomes, obesity, and mode of delivery were related to COVID-19 severity in pregnant patients. However, there was no effect generally on the adverse fetal outcomes except for jaundice and birth weight.

## 1. Introduction

COVID-19, first identified in December 2019, is an infectious disease caused by the novel coronavirus SARS-CoV-2, primarily characterized by respiratory symptoms, but it can also affect other organ systems and lead to severe illness. As of March 2024, over 774 million confirmed cases of COVID-19 and over 7 million deaths have been reported globally [1]. It continues to be a global health concern, with ongoing research efforts aimed at understanding the latest updates in its epidemiology, transmission dynamics, clinical manifestations, and potential interventions. In the United Arab Emirates (UAE), from January 2020 to June 2023, there have been over 1 million confirmed cases of COVID-19 with more than 2000 deaths reported to WHO. Pregnant women represent a unique population that necessitates comprehensive investigation into the impact of COVID-19 infection on their health, obstetric outcomes, and the potential implications for maternal and fetal well-being. A cohort study of 510 pregnant women with COVID-19 infection in sub-Saharan Africa in 2022 showed high rates of intensive care unit (ICU) admission, oxygen supplementation, and death [2]. Pregnant women, their fetuses, and infants are considered the most vulnerable members of society during an infectious disease outbreak. COVID-19 infection in pregnant women poses multiple risks due to changes in their immune system, increased physiological stress, higher chances of preterm birth, preeclampsia, and potential transmission to the fetus [3, 4]. In addition, their upper respiratory tract is swollen with high levels of estrogen and progesterone, and lung levels are limited; therefore, pregnant women are prone to such diseases [4]. Also, changes in physiological adaptation during pregnancy (e.g., increased diaphragm level, increased oxygen consumption, and edema of the mucous membranes of the respiratory tract) cause intolerance to hypoxia [5]. It should be noted that the potential risks of cytokine storm due to infection of pregnant women may cause severe complications and even death [6]. In a case study, researchers observed that infected pregnant women exhibited leukocytosis, lymphopenia, an increased neutrophil ratio, elevated d-dimer levels, elevated C-reactive protein (CRP), and raised lactate dehydrogenase (LDH) [7]. Studies on vertical transmission found positive RT-PCR tests after fetal miscarriages at 19 weeks and fetal deaths at 22 weeks, with the virus detected in placental and umbilical cord biopsies [4]. Another study revealed increased fear and low well-being among pregnant women due to COVID-19, potentially impacting their mental health and pregnancy [8]. A systematic review reported various neonatal outcomes, including stillbirth, preterm birth, fetal distress, and low birth weight [9, 10]. Other studies mentioned premature rupture of membrane, cytokine storm, lung injury, and placental hypoxia [11]. In the UAE, limited studies showed low transmission risk from infected mothers to neonates, with only a few cases testing positive [12, 13]. According to the Ministry of Health and Prevention and the Department of Health—Abu Dhabi, pregnant women were exempted from COVID-19 vaccination under UAE's national guidelines [14]. Further research on COVID-19 effects on implantation, fetal growth, labor, and neonatal health is recommended [4]. Given the limited data available on how COVID-19 affects pregnant women in the UAE, it is imperative to closely track and document outcomes related to maternal health, pregnancy, fetus, and management. By doing so, it may be possible to prevent transmission of the infection to the fetus and to improve our understanding of the most effective management strategies for pregnant women with COVID-19 infection.

## 2. Materials and Methods

### 2.1. Participants

This retrospective descriptive study was conducted at a tertiary hospital for Obstetrics and Gynecology in Ras Al Khaimah (RAK), UAE. The study included pregnant women who tested positive for COVID-19 infection during the period from April 2020 to September 2021. There were a total of 5103 deliveries during the years 2020 and 2021, data was searched for positive reverse transcription polymerase chain reactions tests of nasopharyngeal swabs. The low number of cases is because the majority of pregnant patients with COVID-19 infection sought care at designated COVID-19 centers and were advised to home-quarantine if asymptomatic. They were only referred to the hospital if they tested positive for COVID-19 during their scheduled checkup, if they had significant signs and symptoms or comorbidities, if they were near term or had history of adverse obstetric outcomes, or if fetal checkup for various reasons was indicated. Our inclusion criteria were pregnant women of any age, presenting in any of the three trimesters, who tested positive during the study period. The included cases were divided according to the National Institutes of Health categorization of COVID-19 severity into four categories: [15]

• **Asymptomatic**: tested positive for COVID-19 PCR test but no symptoms,
• **Mild**: patients have symptoms of COVID-19 infection without breathlessness or abnormal radiological findings,
• **Moderate**: patients show signs of lower respiratory disease or imaging indicating that and their oxygen saturation at room air is ≥ 94%,
• **Severe**: patients with oxygen saturation < 94% on room air, arterial partial pressure of oxygen to fraction of inspired oxygen (PaO2/FiO2) < 300 mm Hg, respiratory rate > 30 breaths/min, or lung infiltrates > 50%.
• **Critical**: patients with multiorgan failure, acute renal failure, and septic shock. We added the patients with critical severity with severe cases due to a very low number of cases.

They were further classified into two groups: nonhospitalized (which included asymptomatic and mild cases—*n* = 38) and hospitalized (which included moderate and severe cases—*n* = 17) and data were checked for the hospitalization status of each of the four categories. The minimal estimated sample size is 39 patients, calculated based on a previous study [16].

This study was approved by the Ministry of Health and Prevention Research Ethics Committee/RAK Subcommittee, reference MOHAP/REC/2021/49-2021-UG-M.

### 2.2. Data Collection

Data were collected from the medical electronic record system “Wareed” available in the hospital by the data collection sheet (see Supporting Information [available here]) and included major segments like demographic details, pregnancy details, obstetric and gynecological history, medical history, surgical history, hospital course, COVID-19 infection course, laboratory and radiological results, treatment given, and fetal outcomes at delivery and a week after delivery and postpartum details only during admission until they were discharged, after discharge follow-up was done in public health centers.

### 2.3. Data Analysis

Data were analyzed using SPSS V.26 (IBM Corporation). Continuous variables (age, body mass index [BMI], etc.) were tested against hospitalized/nonhospitalized status. All categorical variables were also tested against hospitalized/nonhospitalized status. The data were compared using Fisher's exact test due to the small number of our sample for categorical data and *t*-test where applicable. It is presented as percentages of the total number of cases in each group (nonhospitalized and hospitalized). A *p* value < 0.05 was considered statistically significant. Missing data were mentioned as missing in the SPSS calculation for maternal adverse outcomes, maternal BMI, comorbidities, laboratory investigations and cases removed in the case of fetal adverse outcomes, and mode of delivery calculations.

## 3. Results and Discussion

### 3.1. Demographic Details

The demographic characteristics and comorbidities of nonhospitalized and hospitalized pregnant women with confirmed COVID-19 infection are shown in Table 1. The mean age was 31 ± 6 years, with the majority of the nonhospitalized and hospitalized patients in the middle-age group 24–34 (60.5% and 52.9%, respectively) (*p*=0.009). Thirty-one patients were Emirati. The mean BMI was 32.4 ± 7 kg/m^2^. There was a significant association between BMI and hospitalization (*p*=0.018). On the other hand, as shown in Table 1, 89% of the hospitalized pregnant women had concurrent comorbidities, in comparison to 74% of the nonhospitalized women. The most common comorbidities identified among our cohort were obesity (54.5%), diabetes mellitus (DM) Type 2 (20%), anemia (18.2%), and gestational diabetes mellitus (GDM) (14.5%). The difference in frequency of DM among hospitalized and nonhospitalized patients showed a trend toward significance (*p*=0.0758). Interestingly, obesity was reported in 44.7% and 76.5% in nonhospitalized and hospitalized patients, respectively, and this difference was significant (*p*=0.0411). Finally, comorbidities that were present in one patient only were grouped under “other diseases” which included hypertension, sickle cell trait, glucose-6-phosphate dehydrogenase (G6PD) deficiency, cardiac problem, thrombocytopenia, Crohn disease, peptic ulcer disease, and RH-isoimmunization.

Most of the patients presented in their third trimester accounting for 47.3% in the nonhospitalized and 21.8% in the hospitalized groups. The mean gestational age (GA) of the cohort was 30.5 ± 10.6. In addition, it was revealed that the mean GA at time of diagnosis was 30 ± 2 and 31 ± 2 weeks for nonhospitalized and hospitalized patients, respectively. However, the relation between GA and hospitalization status was insignificant (*p*=0.733). The mean (SD) of gravida and parity was 4 + 2 and 3 + 2, respectively.

### 3.2. Clinical Features

In the nonhospitalized group, fever was the most common symptom (26%) and sore throat was the least common (3%), whereas in the hospitalized group, cough was the most common symptom (94%) and diarrhea the least (6%). Notably, breathlessness was exclusively reported by the hospitalized patients (76%). Further analysis revealed significant association between hospitalization status and fever, flu-like symptoms (*p*=0.005), sore throat (*p*=0.025), limb/joint pain (*p*=0.026), and breathlessness (*p* < 0.001). Findings are summarized in Table 2.

### 3.3. Laboratory and Radiological Findings

The laboratory findings of the study population are demonstrated in Table 3. Only peak/lowest values were taken. Notably, investigations of CRP, procalcitonin, ferritin, and D-dimer were mostly done for the hospitalized patients, hence the high standard error means. The differences between the mean CRP, hemoglobin, and platelets in nonhospitalized and hospitalized patients were significant (*p*=0.005, *p*=0.049, and *p*=0.011, respectively), while the LDH showed a trend toward significance (*p*=0.053). All other laboratory findings were insignificant.

Chest X-ray was done for 10 (8%) of the hospitalized pregnant patients. The findings retrieved from the electronic medical records showed an image of bilateral lung opacities of ground-glass appearance in 9 patients while one moderate case had a right lung ill-defined basal opacity. Furthermore, 6 chest CT scans were done (10.9%), 5 of which showed bilateral basal and peripheral opacities in lung fields with bilateral extensive multifocal ground glass opacity at upper and lower lobes of both lungs. One moderate case showed bilateral mild pleural effusion with bilateral basal mild atelectasis.

### 3.4. Maternal Outcomes

**Figure 1:**
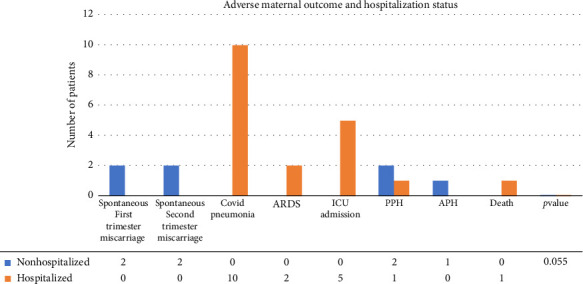
Adverse maternal outcomes and hospitalization status of COVID-19 patients. APH, antepartum hemorrhage; ARDS, acute respiratory distress syndrome; ICU, intensive care unit; PPH, postpartum hemorrhage.

Overall, there were 43 deliveries reported from 55 COVID-19 pregnant women, 4 abortions, and 8 unknown delivery status. Out of the deliveries, 11 were preterm. Adverse maternal outcomes were more frequently reported among the hospitalized patients compared to the nonhospitalized patients (73.3% and 37.9%, respectively, *p*=0.055), which included COVID-19 pneumonia (*n* = 10, 18.2%), acute respiratory distress syndrome (ARDS) (*n* = 2, 3.6%), and ICU admission (*n* = 5, 9.1%). Only one case of maternal mortality was recorded among the hospitalized group. This patient was a 31-year-old woman with morbid obesity. She delivered by emergency C-section due to a previous C-section and acute respiratory distress as a complication of COVID-19. Subsequently, and upon the request of her family, she was transferred to another hospital where her condition deteriorated. She developed COVID-19 complications including ARDS, septicemia, acute renal failure, and multiple organ damage, and passed away approximately a month after delivery. Another case in the hospitalized group involved a 25-year-old woman who was admitted at 35 weeks of pregnancy. She delivered by emergency C-section due to acute respiratory distress as a complication of COVID-19, for which she also needed intubation. A chest X-ray showed a typical picture of viral pneumonia. The baby was a healthy girl with APGAR scores of 9, 10, and 10 at 1, 5, and 10 min, respectively. The patient improved and was discharged in stable condition. Both cases were admitted at the beginning of the pandemic crisis. Other adverse outcomes reported included miscarriages in the first trimester (*n* = 2, 3.6%) and second trimester (*n* = 2, 3.6%), postpartum hemorrhage (PPH) (*n* = 3, 5.5%), and antepartum hemorrhage (APH) (*n* = 1, 1.8%) (Figure 1). All the 55 pregnant patients included in the study were not vaccinated for COVID-19 as they were exempted from vaccination according to the UAE's national guidelines applied by the Ministry of Health and Prevention and Department of Health—Abu Dhabi [14].

### 3.5. Mode of Delivery and Hospitalization

The mode of delivery varied significantly in nonhospitalized versus hospitalized pregnant women (Table 4), with emergency cesarean section being the most common mode of delivery in hospitalized patients (*n* = 11, 5.6%, *p*=0.0007). Elective lower segment cesarean section deliveries were predominantly higher in hospitalized patients as opposed to nonhospitalized patients. Spontaneous vaginal delivery was the most common delivery mode in nonhospitalized patients (*n* = 18, 64.3%, *p*=0.0097). In addition, 5 of the nonhospitalized patients (17.9%) had induced vaginal delivery (*p*=0.4029). In conclusion, the nonhospitalized patients underwent the physiological path of delivery in comparison with the hospitalized women who had to face riskier modes of delivery.

### 3.6. Fetal Outcomes

With respect to the fetal outcomes, the full data of only 43 neonates were retrieved from the electronic medical records, 8 delivery records were not available due to patients delivering in other hospitals.

Adverse outcomes were reported in 10/28 (35.7%) neonates born to the nonhospitalized patients compared to 8/15 (53.3%) neonates born to the hospitalized patients. However, this difference was statistically insignificant (*p*=0.521), except for jaundice which was significant (*p*=0.0146). From 43 deliveries, 26 were females and 17 were males. The average birth weight of all newborns was 3.1 ± 0.46 kg. There was a missing birth weight of one of the 43 deliveries as the mother delivered in another hospital and the birth weight could not be retrieved. Notably, babies born to hospitalized patients showed a significantly lower mean neonatal birth weight of 2.88 + 0.44 kg compared to 3.21 + 0.46 kg of the babies born to nonhospitalized patients (*p*=0.028). This could be due to the fact that there were more premature babies born to the hospitalized patients. The overall mean of APGAR scores at 1 and 5 min was 8.61 ± 1.3 and 9.63 ± 1.04, respectively, which showed nonsignificant relation to the hospitalization status (*p* value = 0.908, 0.786, respectively) (Table 5).

The most frequently reported adverse fetal outcome was admission into the neonatal intensive care unit (NICU). Of the 6 admitted neonates, 4 were preterm, in which one of them had various adverse outcomes including neonatal sepsis and jaundice and his mother was in the hospitalized group. Also, there is one meconium-stained liquor neonate, one had RDS and meconium liquor as well. Only one neonatal death was recorded in our cohort of a female baby, delivered at 32 weeks and 6 days to a nonhospitalized patient by emergency cesarean section. She had low APGAR scores 1, 4, and 4 at 1, 5, and 10 min; her chest X-ray revealed pleural effusion and later expired due to hydrops fetalis. The other adverse fetal outcomes are described in Table 5.

### 3.7. Treatment

Thirty-four pregnant women with COVID-19 infection received treatment, ranging from broad-spectrum antibiotics to COVID-19-related medications and oxygen support. Most of the nonhospitalized patients received supportive medications like dexamethasone, enoxaparin, and ipratropium bromide, whereas hospitalized patients received antiviral drugs and oxygen support. Noninvasive oxygen support was provided to 10 patients, while only one received invasive oxygen support using endotracheal intubation. Common noninvasive oxygen support methods included face mask, high flow nasal cannula, and noninvasive CPAP. The medications prescribed to the patients are listed in Table 6.

## 4. Discussion

The growing body of evidence suggests that COVID-19 during pregnancy poses significant risks to the health of both mothers and fetuses, highlighting the importance of preventative measures and early monitoring for pregnant individuals. There are limited studies conducted in the UAE regarding COVID-19 effects on pregnancy and on immediate fetal outcomes. Thus, the current study focused on observing these parameters retrospectively from a tertiary center in RAK, UAE. Our study took place during the period from April 2020 to September 2021. As per the UAE law, patients who were asymptomatic or had mild symptoms were advised to isolate at home, as opposed to hospital or quarantine camp admission for moderate, severe, and critical COVID-19 infection.

In our study, most of the patients were classified into nonhospitalized (which included asymptomatic and mild cases—*n* = 38) and hospitalized (which included moderate and severe cases—*n* = 17) which is comparable to other studies [17–20]. In addition, higher number of COVID-19-infected pregnant patients were in their third trimester (69.1%), of which 21.8% were hospitalized, which matches the findings of previous studies [21–24]. This could be explained by the active inflammatory response in the mother during the first and second trimesters, so getting an infection is less likely as compared to third trimester period [25, 26]. It was noted that the GA at time of COVID-19 diagnosis had no effect on the disease severity and hospitalization status. The study revealed a significant correlation between hospitalization status and maternal age (*p*=0.009). The majority of the patients were in the middle-age group (24–34 years), which may explain why most patients were asymptomatic and few were admitted to hospital with moderate–severe condition. Similarly, Epelboin et al. showed that old age is associated with severe COVID-19 infection [27].

In regard to comorbidities and severity of the COVID-19 infection, the present study found that BMI (obesity and morbid obesity) had the most significant effect on the severity/hospitalization status of the patients, and it was also the most frequent comorbidity among both groups, which is in line with other studies [19, 20, 27, 28]. Obesity is said to cause chronic inflammation and inhibition of viral killing by delaying IL-1 and IL-3 reactions. A case report in Iran noted the death of one pregnant woman with morbid obesity and COVID-19 infection, which is thought to be due to obesity making the infection severe [28]. In our study, we encountered one maternal death of a patient with BMI of 40 kg/m^2^ who deteriorated after delivery. Based on the patient's family request, she was transferred to another hospital, where she expired. Anemia and DM accounted for 15.8% and 13.2%, respectively, as next most common comorbidities in the nonhospitalized group in comparison with DM being second most common in the hospitalized group (35.3%). In a similar study conducted in Dubai, DM was the commonest comorbidity and in another, DM as a preexisting disease was also associated with COVID-19 severity as in comparison to our study it showed a trend toward significance (*p*=0.0758) [27, 29]. Ultimately, our study did not reveal significant association between having comorbidity and hospitalization status other than obesity (Table 1).

Most previous similar studies documented fever, cough, and dyspnea as the most frequent presenting clinical features in pregnant women with COVID-19 infection. Additionally, fatigue, myalgia, headache, runny nose, and diarrhea were also observed in these studies [3, 11, 18, 21, 24, 29–32]. Another study also noted that most patients were asymptomatic [3]. These findings are in line with our data except that diarrhea was the least frequent symptom. In agreement with our results, previous studies described CT chest findings found in all COVID-19-positive cases, and they most included patchy ground-glass opacity with or without consolidation, and pleural effusion [18, 24, 31].

As reported by other studies, COVID-19-infected pregnant women had elevated neutrophils and CRP and reduced lymphocytes correlating with severity. Our results demonstrated higher WBC and CRP (*p*=0.005) and lower lymphocyte count in hospitalized COVID-19 pregnant patients compared to the nonhospitalized ones. LDH and ferritin levels showed no significant difference between pregnant and nonpregnant COVID-19-infected women [22, 29, 33–35], aligning with our study. Pregnancy induces hypercoagulability, compounded by COVID-19. While a previous study reported significantly elevated D-dimer in infected pregnant women, our study found a nonsignificant difference possibly due to a smaller sample size. The findings emphasize monitoring specific parameters in pregnant women with COVID-19 for better risk assessment [29].

The impact of COVID-19 on maternal health during pregnancy has been a significant concern since the start of the pandemic. COVID-19-positive pregnant women were more likely to have an intervention during their pregnancy, specifically to be delivered by means of cesarean section, especially the severe cases [17, 19, 23, 36]. These findings were consistent with the current study as cesarean delivery was found to be significant (*p* < 0.001) in hospitalized patients when compared to nonhospitalized, and most of them were emergency rather than elective. Furthermore, nonhospitalized pregnant women were more likely to be delivered through induction or more commonly spontaneous (*p*=0.002). Notably, the adverse maternal outcomes were witnessed more in hospitalized patients than the nonhospitalized pregnant patients. In our hospitalized group, there were 11 preterm deliveries which is supported by another study that showed increased rates of preterm delivery with symptomatic COVID-19, as compared to noninfected women [10, 18, 37]. Nonetheless, another study reported no increases in the risks of spontaneous preterm birth in pregnant COVID-19 patients [38]. Our study revealed that COVID-19 pneumonia was the prevailing adverse maternal outcome, with 10 pregnant women being hospitalized for assessment and treatment, of those, 5 required admissions to the ICU. This corresponds to another investigation that found a range of severe pneumonia rates among the cases, from 0% to 14%, with most of them needing admission to the ICU [31]. Another study revealed that severe COVID-19 pneumonia accounted for 6 out of 22 emergency LSCS procedures [29]. Various research studies have indicated that expectant mothers who contract COVID-19 may face a higher likelihood of pregnancy-related complications like PPH and APH [39]. The exact reason behind this correlation is still unclear, though some experts have suggested that the virus could aggravate blood clots, resulting in bleeding complications. It should be noted, however, that not all pregnant COVID-19 patients develop such issues, and most cases of PPH and APH are not related to the virus. According to a methodical analysis, the infection of COVID-19 among expectant mothers was linked to a higher probability of experiencing APH [40]. In our study, we identified three instances of PPH and one incident of APH in pregnant women with COVID-19. The exact mechanism behind the potential link between preeclampsia and COVID-19 infections is still not known and is currently being studied. However, it is believed that the immune response and inflammation caused by COVID-19 could potentially exacerbate the symptoms of preeclampsia in pregnant women, leading to a higher risk of adverse outcomes [39, 41]. The relationship between the two conditions could be explained by the fact that both of them are associated with inflammation and endothelial dysfunction [42]. Recent studies indicate that pregnant women who contract COVID-19 may be at a higher risk of developing cardiomyopathy and other cardiovascular complications including myocarditis, pericarditis, heart failure, and arrhythmias, especially if associated with other comorbidities [43–45]. Therefore, early identification and management of complications are crucial to improve maternal and fetal outcomes.

The relationship between COVID-19 infection and neonatal birth weight remains a complex issue, with conflicting findings reported in the literature. While some studies have found no significant difference in birth weight between neonates born to mothers with and without COVID-19 infection, others have reported lower birth weights among neonates born to infected mothers. For instance, studies conducted in New York City and Italy have reported lower mean birth weights among neonates born to mothers with COVID-19 infection. Our study also revealed that COVID-19-infected hospitalized pregnant patients were more likely to give birth to lighter newborns than nonhospitalized mothers, with an average birth weight of 2.81 kg compared to 3.14 kg. This could be partly due to the higher incidence of premature births among hospitalized mothers [46, 47]. A study conducted in the UAE also showed that the average birth weight of neonates born to COVID-19-positive mothers was 2.8 kg [29]. However, it is important to note that some studies have found no significant association between COVID-19 infection and neonatal birth weight [20].

COVID-19 infection during pregnancy has been linked to various unfavorable fetal outcomes, such as stillbirth, preterm birth, and fetal distress. Our research demonstrated that at 1 min, the mean APGAR score was 8.61 + 1.3, whereas at 5 min, the mean score was 9.63 + 1.04. Comparatively, another research study found that the APGAR score ranges at both 1 and 5 min were between 7 and 10 [31]. An earlier retrospective study carried out in China revealed that infants born to COVID-19-positive mothers had a lower average APGAR score at 1 min in comparison with infants born to COVID-19-negative mothers (8.14 vs. 9.08). However, there was no significant difference observed in the mean APGAR scores at 5 min between the two groups [48]. A comprehensive systematic review and meta-analysis of 77 studies conducted across multiple countries highlighted a greater occurrence of cesarean delivery and NICU admission in COVID-19-positive pregnant women [49]. Further evidence continues to support the potential risks that COVID-19 infection poses to fetal health during pregnancy [36, 50, 51]. A meta-analysis in 2022 discussed concerns about the possibility of vertical transmission of COVID-19 from mother to baby. Limited studies have been conducted on this topic, and there is conflicting information. A systematic review of 32 studies found that 2% of infected mothers had neonates who tested positive for COVID-19, but in most studies, there was no evidence of vertical transmission. While neonates and children typically do not develop severe COVID-19 and neonatal mortality is rare, caution should still be exercised regarding the possibility of vertical transmission [52]. Our own study aligns with previous research demonstrating that COVID-19 infection during pregnancy is linked to a higher incidence of NICU admission in neonates, as well as a greater risk of preterm birth and fetal distress. The most frequent adverse outcome noticed in our study was NICU admission, with prematurity, respiratory distress, and meconium-stained liquor as the most common reasons for admission, consistent with previous research.

Furthermore, our study identified three cases of neonates in fetal distress, which may be caused by maternal inflammation or other complications related to COVID-19 infection and can lead to placental insufficiency. Lastly, our study reported three small for gestational age (SGA) neonates, a condition that has also been linked to COVID-19 infection during pregnancy. A study conducted in the United States during the pandemic found a significant increase in preterm births, stillbirths, and NICU admissions. However, there was no significant change in other outcomes such as fetal growth restriction or neonatal mortality. The authors suggested that pandemic-related stress and disruptions to healthcare services may have contributed to these negative outcomes [53]. A cohort study in Iraq and Saudi Arabia found that pregnant women with COVID-19 infection are at increased risk for premature births, miscarriages, preterm pre-labor rupture of membranes, and NICU hospitalizations [54, 55].

Several factors have been associated with preterm births in pregnant women with COVID-19, including high BMI, cesarean delivery, nulliparity, and severe COVID-19 symptoms. Furthermore, in cases where pregnant women experienced severe COVID-19 symptoms, their newborns had a higher incidence of respiratory distress syndrome [23]. Overall, the findings of our study are in accordance with the existing literature on the link between COVID-19 infection during pregnancy and adverse fetal outcomes. These findings emphasize the need for early monitoring and preventative measures for pregnant women who are at higher risk of contracting COVID-19. A systematic review and meta-analysis suggest that COVID-19 infection during pregnancy is associated with a higher risk of adverse fetal outcomes such as preterm delivery, maternal mortality, NICU admission, and neonatal death, particularly in women with severe disease and comorbidities. However, the authors noted that the absolute risk of adverse fetal outcomes remains low, and further research is needed to better understand the impact of COVID-19 on pregnancy outcomes [52].

Finally, the management of the pregnant women during COVID-19 infection is very important, as it may contribute to the pregnancy outcomes later. In a study done on pregnant women in Hubei Province, they concluded that the use of long-acting uterotonic agents can reduce the incidence of PPH during surgery, in addition to the sake of the treatment of maternal pneumonia termination of pregnancy in case of severe COVID-19 infection may be beneficial of the mother [56]. COVID-19 management for pregnant women includes level 2/3 critical care, supplemental oxygen, noninvasive ventilation, intubation, extracorporeal membrane oxygenation, antiviral, other specific anti-COVID-19 therapy, iatrogenic delivery, or end due to maternal conceded from COVID-19 [16]. In our study, the nonhospitalized patients mainly received supportive medications like dexamethasone, enoxaparin, and ipratropium bromide, while the hospitalized patients were given stronger medications such as antiviral drugs and oxygen support. The most common drug used was enoxaparin, followed by dexamethasone, hydroxychloroquine, and interferon alpha 1b. During normal pregnancy, unfractionated heparin is preferred at the end of pregnancy for its easy reversibility [17]. Medications like lopinavir/ritonavir and hydroxychloroquine were used as first-line treatment for COVID-19 [29]. A study suggested that antiviral therapies like remdesivir and monoclonal antibodies, corticosteroids for severe cases, and supportive care such as oxygen therapy and fluid management can improve outcomes for COVID-19 patients [57]. Another study found that the most common medication given to pregnant women with COVID-19 was azithromycin, followed by dexamethasone, remdesivir, and heparin [58].

As our study was conducted during the early stages of the pandemic, the side effects of COVID-19 vaccinations on pregnant women were unknown, and therefore they were exempted from vaccination. Recent studies show that mothers vaccinated before and during pregnancy develop antibodies that protect the baby from the virus, reduce the risk of severe COVID-19 infection for the mother during pregnancy, and might also help prevent stillbirths and preterm delivery. Additionally, vaccinating the mother can protect babies less than 6 months old from hospitalization. The WHO even recommends a single-dose vaccination against COVID-19 during pregnancy as pregnant mothers are at a higher risk of getting infected [59, 60].

### 4.1. Study Limitations

The management guidelines of the novel COVID-19 infection were not completely set in 2020, when half our cohort was recruited. Hence, some laboratory investigations such as procalcitonin, D-dimer, LDH, and CRP were only done for severely symptomatic patients. Another limitation was the small sample size available. This could be due to patients' lack of awareness of COVID-19 symptoms and their willingness to report to the hospital for mild flu-like symptoms. Lastly, a few of the patients who presented in early pregnancy with COVID-19 infection could not be followed up later on possibly because they delivered in private hospitals or their home countries. Our access was only limited to pregnant patients' data and immediate newborn complications.

## 5. Conclusions

The current study provided an insight into the maternal and fetal outcomes of COVID-19 infection in pregnant women in tertiary hospital for Obstetrics and Gynecology in RAK, UAE. The study reported obesity as the most significant comorbidity among pregnant women with severe COVID-19 infection. The most common symptoms were fever and flu-like symptoms. Hospitalized patients revealed significantly elevated CRP, low hemoglobin and platelet levels, with lactose dehydrogenase showing a trend toward significance. Overall, the adverse maternal outcomes differed significantly between the studied groups. Hospitalized patients experienced higher rates of pneumonia, ICU admission, ARDS, and merely maternal mortality. Additionally, emergency cesarean section was the predominant mode of delivery reported in the hospitalized patients. Of the adverse fetal outcomes recorded, only jaundice and low birth weight were significantly correlated with the maternal COVID-19 infection severity.

## Figures and Tables

**Table 1:** Demographic characteristics and comorbidities of nonhospitalized and hospitalized COVID-19 patients.

Patients' characteristics and demographic data	Nonhospitalized (*n* = 38)	Hospitalized (*n* = 17)	*p* value
Agea			
18–24	6 (15.8%)	0	
24–34	23 (60.5%)	9 (52.9%)	
35–44	9 (23.7%)	8 (47.1%)	
Age in years, mean ± SDb	30 ± 5.8	35 ± 5.1	0.009^∗∗^
Nationalitya			
UAE	21 (55.3%)	10 (58.8%)	1.000
Non-UAE	17 (44.7%)	7 (41.2%)	
BMI	*n* = 37	*n* = 17	
18.5–24.9	5 (13.5%)	0	
25–29.9	15 (40.5%)	4 (23.5%)	
30–39.9	16 (43.2%)	8 (47.1%)	
> 40	1 (2.7%)	5 (29.4%)	
BMI, kg/m^2^ mean ± SDb	30.6 ± 5.6	36.2 ± 8.3	0.018^∗^
Gestational age at admission in weeks, mean ± SDb	30 ± 12	31 ± 8	0.733
Comorbidities^a^			
None	17 (44.7%)	4 (23.5%)	0.2292
DM	5 (13.2%)	6 (35.3%)	0.0758
Hypertension	0	1 (5.9%)	0.3091
Group B *Streptococcus* (GBS) +	3 (7.9%)	0	0.5445
Obesity	17 (44.7%)	13 (76.5%)	0.0411^∗^
Psychiatric illness	3 (7.9%)	1 (5.9%)	1
Hypothyroidism	3 (7.9%)	1 (5.9%)	1
Anemia	6 (15.8%)	4 (23.5%)	0.4792
Allergy	2 (5%)	2 (11.8%)	0.5795
GDM	4 (10.5%)	4 (23.5%)	0.2351
Other diseases	7 (18.4%)	1 (5.9%)	0.4113

Abbreviations: BMI, body mass index; DM, diabetes mellitus; GDM, gestational diabetes mellitus; UAE, United Arab Emirates.

^a^Fisher's exact test.

^b^Independent *t*-test.

^∗^ *p* < 0.05.

^∗∗^ *p* < 0.01.

**Table 2:** Presenting symptoms of nonhospitalized and hospitalized COVID-19 patients.

Symptoms (*n* (%))	Nonhospitalized (*n* = 38)	Hospitalized (*n* = 17)	*p* value (Fisher's exact test)
Fever 22/40%	10 (26%)	12 (71%)	0.003
Cough 25/45.5%	9 (24%)	16 (94%)	< 0.001
Sore throat 5/9.1%	1 (3%)	4 (24%)	0.025
Headache 6/10.9%	4 (11%)	2 (12%)	1.000
Lethargy 10/18.2%	2 (5%)	8 (47%)	< 0.001
Limb/Joint pain 7/13.7%	2 (5%)	5 (29%)	0.026
Diarrhea 3/5.5%	2 (5%)	1 (6%)	1.000
Breathlessness 13/23.6%	0	13 (76%)	< 0.001
Flu-like symptoms10/18.2%	3 (8%)	7 (41%)	0.005

**Table 3:** Laboratory findings of nonhospitalized and hospitalized COVID-19 patients.

	Nonhospitalized (*N* = 38)		Hospitalized (*N* = 17)		*p* value
	*N*	Mean ± standard error	*N*	Mean ± standard error	
Hgb (gm/dL)	35	11.3 ± 0.2	17	10.5 ± 0.4	0.049^∗^
D-dimer (mg/L FEU)	6	2.3 ± 0.5	15	1.8 ± 0.3	0.357
Procalcitonin (ug/L)	3	0.097 ± 0.068	14	0.8 ± 0.7	0.360
CRP (mg/L)	7	16.3 ± 5.1	17	72.0 ± 11.4	0.005^∗∗^
Ferritin (ng/mL)	6	78.5 ± 35.5	12	91.7 ± 35.4	0.818
Platelets (x10^3^/mcL)	36	241.2 ± 12.8	17	181.8 ± 18.4	0.011^∗^
WBC (x10^3^/mcL)	36	7.9 ± 0.4	17	8.5 ± 1.6	0.782
Absolute lymphocytes (×10^3^/mcL)	36	15.5 ± 1.9	16	14.3 ± 2.5	0.735
PTT (seconds)	15	32.4 ± 0.8	14	34.8 ± 1.2	0.110
INR (×10^3^/mcL)	10	0.99 ± 0.02	16	1.1 ± 0.1	0.297
LDH	2	219.5 ± 16.5	10	324.2 ± 40.2	0.053

Abbreviations: CRP, C-reactive protein; Hgb, hemoglobin; INR, international normalized ratio; LDH, lactate dehydrogenase; PPT, partial thromboplastin time; WBC, white blood cells.

^∗^ *p* < 0.05.

^∗∗^ *p* < 0.01.

**Table 4:** Association of mode of delivery and the hospitalization status.

	Nonhospitalized (*n* = 28, 65.1%)	Hospitalized (*n* = 15, 34.9%)	*p* value (Fisher's exact test)
Spontaneous vaginal delivery	18 (64.3%)	3 (20%)	0.0097^∗∗^
Induced vaginal delivery	5 (17.9%)	1 (6.7%)	0.4029
Cesarean section	5 (10.4%)	11 (5.6%)	0.0007^∗∗∗^
• Emergency cesarean section	• 4	• 7	
• Elective lower segment cesarean section	• 1	• 4	

^∗∗^ *p* < 0.01.

^∗∗∗^ *p* < 0.001.

**Table 5:** Association of fetal outcomes with hospitalization status of COVID-19 patients.

Fetal outcomes	Nonhospitalized (*n* = 28, 65.1%)	Hospitalized (*n* = 15, 34.9%)	*p* value
Birth weightb	3.21 ± 0.46 kg	2.88 ± 0.44 kg	0.028^∗^
APGAR at 1 min mean ± SDb	8.59 ± 1.58	8.64 ± 0.5	0.908
APGAR at 5 min mean ± SDb	9.67 + 1.21	9.57 + 0.65	0.786
No adverse fetal outcomes^a^	12 (42.9%)	3 (20%)	0.1864
Small for gestational agea	3 (10.7%)	0	0.5406
Meconium-staineda	3 (10.7%)	0	0.5406
Fetal distress during labora	1 (3.6%)	2 (13.3%)	0.2751
Respiratory distress syndromea	1 (3.6%)	1 (6.7%)	1
Prematurea	1 (3.6%)	3 (20%)	0.1143
Jaundicea	1 (3.6%)	5 (33.3%)	0.0146^∗∗^
NICU admissiona	5 (17.9%)	1 (6.7%)	0.4029
Neonatal deatha	1 (3.6%)	0	1

*Note:* Please note that the same neonate may have two or more outcomes.

^a^Fisher's exact test.

^b^Independent *t*-test.

^∗^ *p* < 0.05.

^∗∗^ *p* < 0.01.

**Table 6:** Treatment modalities of the COVID-19 patients.

Treatment	Number of patients who received it (*n* = 34)
Oxygen support	11
Noninvasive	10 (29.4%)
Invasive	1 (2.9%)
Covid-19-related medications	23
Enoxaparin	25 (73.5%)
Hydroxychloroquine	7 (20.6%)
Dexamethasone	8 (23.5%)
Favipiravir	1 (2.9%)
Lopinavir–ritonavir	1 (2.9%)
Interferon alpha 2b	7 (20.6%)
Remidesavir	5 (14.7%)
Ritonavir	2 (5.9%)
Ipratropium bromide	2 (5.9%)
Budesonide	2 (5.9%)
Monoclonal antibody	5 (14.7%)
Antibiotics	23 (67.6%)

## Data Availability

Data are contained within the original article.

## Supporting Information

A data collection sheet which was used as a guide to collect data from the Hospital's electronic record system is available in the “Supporting information” file.
