# Supplementary material for: COVID-19 and Pregnancy Outcomes: A Descriptive Study From a Tertiary Hospital in Ras Al Khaimah, UAE
Source: Obstet Gynecol Int. 2024 Dec 3;2024:5252919. doi: 10.1155/ogi/5252919 (PMC11631290; doi:10.1155/ogi/5252919)
Supplement: Supporting Information — Additional supporting information can be found online in the Supporting Information section. [file 5252919.f1.pdf]

The following data collection sheet which was used as a guide to collect data from the Hospital's electronic record system is available in the "supplementary material" file.

Title of the project:
COVID-19 and Pregnancy Outcomes: A Descriptive Study From a Tertiary Hospital in Ras Al Khaimah, UAE.

Patient's File No: Date of Admission:

Age/DOB: Sex: ☐ M ☐ F Date of Discharge:

Nationality: Weight: Height:

Complaints on Admission:

Medical History:

☐ **Diabetes mellitus**	☐ **Chronic kidney disease**	☐ **Hypertension**
☐ **Malignancy**	☐ **Immunocompromised state**	☐ **Sickle cell disease**
☐ **COPD**	☐ **Asthma**	☐ **Heart Conditions**
☐ **Cerebrovascular disease**	☐ **Cystic fibrosis**	☐ **Pregnancy**
☐ **Neurologic conditions**	☐ **Pulmonary fibrosis**	☐ **Thalassemia**
☐ **Overweight**	☐ **Liver disease**	☐ **Obesity**
☐ **Severe Obesity**	☐ **Other :..**	

Medication History:

☐ **Anti-hypertensive**	☐ **Anti-psychotic**	☐ **Anti-diabetic**
☐ **Anti-osteoporotic**	☐ **NSAIDs**	☐ **Anti-platelet**
☐ **Analgesic**	☐ **Anti-coagulant**	☐ **Anti-depressant**
☐ **Anti-asthmatic**	☐ **Other :..**	

Social History:

Tobacco use	☐ Current smoker	☐ Former smoker	☐ Non-smoker
Alcohol use	☐ Alcoholic	☐ Non-alcoholic	

Onset History:

Symptoms at Disease Onset	☐ Fever	☐ Chills	☐ Cough
	☐ Shortness of breath	☐ Chest pain	☐ loss of smell
	☐ Sputum production	☐ Diarrhea	☐ Nausea
	☐ vomiting	☐ Headache	☐ Muscle pain
	☐ abdominal pain	☐ loss of test	☐ Malaise C
	☐ Nasal congestion	☐ Sore throat	☐ Other:..
Testing Method for COVID-19 Diagnosis	☐ SARS-COV-2 PCR	☐ Other:	

Known Exposure to COVID-19	☐ Yes, (Household/Community/Healthcare/Occupational/Travel)<br>☐ No
COVID Status and Progression:<br>Vaccination status	
Vital Signs	
Severity of COVID-19	☐ Mild ☐ Moderate ☐ Severe/ Critical
Duration of hospital stay (days)	
Investigations:	
Radiology	Chest X ray/CT scan/Ultrasound findings if any
Chemistry and Hematology	Blood grouping, Rh factor, d-dimer and others (Coagulation profile and important CBC components and important inflammatory markers
Microbiology	
Treatment:	
COVID-19 Specific Medications	
Other Medications<br>**Obstetrics& Gynaecology history**	
**Maternal outcomes**	Delivery mode, PPH, PROM, preterm, prolonged labor, placental abnormalities, problems, delay in placental delivery, chorioamnionitis, PIH, GDM, maternal death, respiratory distress, pneumonia, cytokine storm, pregnancy termination or miscarriage, antepartum haemorrhage
**Fetal outcomes**	Stillbirth, AGA, SGA, LGA, birth weight, APGAR score, prematurity, gestational age, NICU admission, fever, breastfeeding, congenital anomalies, death, IUGR; jaundice, meconium stained or aspiration, infected with COVID-19, fetal distress.
Adverse drug reactions	☐ Yes ☐ No

Recovered from COVID-19 / Death	☐ Recovered ☐ Death
